# Psychosocial ergonomics of the workplace of medical staff during the COVID-19 pandemic in three risk’s dimensions: working hours, violence and the use of psychoactive drugs—a prospective pilot study

**DOI:** 10.3389/fpubh.2023.1199695

**Published:** 2023-07-04

**Authors:** Łukasz Rypicz, Paweł Gawłowski, Izabela Witczak, Alicja Humeńczuk-Skrzypek, Hugh Pierre Salehi, Anna Kołcz

**Affiliations:** ^1^Division of Public Health, Department of Population Health, Faculty of Health Sciences, Wrocław Medical University, Wrocław, Poland; ^2^Center for Medical Simulation, Wrocław Medical University, Wrocław, Poland; ^3^Department of Public Health, University Clinical Hospital, Wrocław, Poland; ^4^Department of Industrial Engineering, The Ohio State University, Columbus, OH, United States; ^5^Ergonomics and Biomedical Monitoring Laboratory, Department of Physiotherapy, Faculty of Health Sciences, Wrocław Medical University, Wrocław, Poland

**Keywords:** ergonomics, medical staff, occupational risk, workplace, occupational environment

## Abstract

**Introduction:**

Workplace ergonomics should also be considered in the context of psychosocial factors affecting the worker, which have a real impact on occupational risk. The present study examined psychosocial risk factors in medical personnel in three domains: working hours, violence and substance abuse.

**Methods:**

The purpose of the present study is to assess the current state of psychosocial ergonomics of medical personnels by measuring occupational risks in the domains of: working hours, violence and psychoactive substance abuse. The survey is consisted of two parts: socio-demographic information of participants and participants’ assements of psychosocial risk factors.

**Results:**

In more than half of the respondents (52%), increased risk was identified in the domain of working hours. Nearly half of the respondents (49.6%) have an identified high risk in the domain of violence, and more than half of the respondents (52%) are at high risk in the domain of psychoactive substance abuse.

**Discussion:**

Our findings show that the present psychosocial ergonomics of the Polish health system must be improved. The COVID-19 pandemic has been a compelling test to assess the current state. Our findings highlighted the fact that HCWs often worked overtime and that many cases of workplace violence and substance abuse were reported.

## 1. Introduction

Poor psychosocial ergonomics at work should be considered a safety hazard because it has a negative impact on the mental health of workers. In many instances, the ergonomics of the workplace is poor in health care. Adverse ergonomics can be detrimental to the mental health of health care workers (HCWs). Given the high and unavoidable work stress of HCWs, it is important to reduce stress from imperfect work ergonomics to improve the mental health of workers. To assess the psychosocial ergonomics of HCWs, we investigated psychosocial risk factors in three domains: hours of work, experience of workplace violence, and substance abuse.

We considered working hours as a major factor, since the long and exhausting working shifts of HCWs make them more exposed to stresses caused by imperfect work workplace ergonomics (1–4).

Workplace violence against medical personnel has a negative effect on workers mental health, exert psychological pressure on them, and can result in workplace absenteeism, job resignation (5–8). Workplace violence is complex problem which can impact different practitioners and can be caused by various risk factors including, high demand for health services, shortages of HCWs, long waiting times for health services, limited interpersonal trust, unrealistic patient expectations, and medical errors (9, 10).

There have been concerns about substance abuse, including alcohol, nicotine, and particularly psychoactive drugs that can alter the cognitive treatment, mood, and emotions of health care workers (11, 12). Predisposing factors associated with substances abuse among HCWs are complex and includes, dealing with pain and death of patients, workload, mental and physical exhaustion, low salaries, as well as access to psychotropic drugs (13). Since substance abuse among health care workers is a mechanism for coping with stress at work, the measurement of the extent of substance abuse among health care workers is an indicator of ergonomics in the workplace (14).

While many medical workers have faced unfavourable psychosocial ergonomics at work, the SARS-CoV-2 epidemic has exacerbated the problem. Factors such as fear of infection, forced overtime work, fatigue, and family isolation affected the mental health of medical workers during the outbreak which might engender risky behaviors such as substance use (15–17).

To understand the preparedness of our healthcare ergonomics in relation to epidemics, we evaluated occupational risks in the domains of which are outcomes of poor workplace psychosocial ergonomics: working hours, work place violence experience, and abuse of psychoactive drugs.

## 2. Materials and methods

### 2.1. Participants and settings

The survey was conducted between November 1, 2021 and December 31, 2021 during the COVID-19 pandemic. The survey was conducted in an online format, by using the electronic survey platform www.webankieta.pl.

The survey was distributed to medical staff of the Wroclaw University of Medical Sciences including physicians, dentists, nurses, midwives, paramedics, and physiotherapists. Potential participants were given a link to the survey through their medical groups and social media. Completion of the survey was voluntary and data was collected anonymously. Participants could withdraw anytime. An IP address filtering (a numerical identifier given to a network interface) was used to avoid collecting duplicate responses from a participant.

The inclusion criterion for this study was being an active healthcare provider at the time of the survey, i.e., November–December 2021, during the COVID-19 pandemic. There were 143 potential participants in this study. From all, 18 surveyees submitted their response incomplete which were excluded from statistical analysis.

### 2.2. Research tool

The survey consists of two parts:

a) socio-demographic information about participants.
b) Participants’ assessments of psychosocial risk factors.

The psychosocial risk factors section goes over three major themes, and each theme consists of 15 questions which are adopted from the European Commission’s guide to health and safety risks in the healthcare sector (18):

a) Working hours.
b) Violence.
c) Abuse of psychoactive substances.

Surveeyes chose either “applicable” or “not applicable” in response to psychosocial risk factors’ questions. We used the aggregated scores to asses the severity of psychosocial risks. The risk levels were defined as follows:

a) No risk (1–5 marked answers “applicable”)—the need to take action on individual elements.
b) Increased risk (6–10 marked “applicable” answers)—structural and control analyses are recommended.
c) High risk (11–15 marked “applicable” answers)—need for urgent structural and control analyses.

For the purposes of this study, the following definition of “external” workplace violence was adopted: insults, threats, and physical or psychological aggression from people outside the organization, including customers (patients), which is directed against the person doing the work and threatens his or her health, safety or well-being - including those with racial or sexual motivation (18).

### 2.3. Ethical considerations

The study was carried out in accordance with the tenets of the Declaration of Helsinki and guidelines of Good Clinical Practice (World Medical Association, 2013).

Written information about the study was provided as an introduction to the survey, with an emphasis on the voluntary and anonymous nature of participation and its guaranteed confidentiality. By answering the questionnaire, participants gave their consent to participate in the study. The research project was approved by the independent Bioethics Committee at the Wroclaw Medical University (No. KB–613/2021).

### 2.4. Statistical analysis

In the study, the analysis of quantitative variables (i.e., expressed by number) was carried out by calculating the mean, standard deviation, median and quartiles. The analysis of qualitative variables (i.e., not expressed by number) was carried out by calculating the number and percentage of occurrences of each value. Comparison of the values of quantitative variables in the two groups was performed using the Mann–Whitney test. Comparison of the values of quantitative variables in three or more groups was performed using the Kruskal–Wallis test. When statistically significant differences were detected, post-hoc analysis was performed with Dunn’s test to identify statistically significantly different groups. Multivariate analysis of the effect of multiple variables on a quantitative variable was performed using linear regression. The results are presented in the form of regression model parameter values with 95% confidence intervals. The analysis assumed a significance level of 0.05. So, all *p*-values below 0.05 were interpreted as indicating significant relationships. The analysis was performed in the R program, version 3.6.1 (R Core Team, 2019) (19).

## 3. Results

The study group was gender-diverse: 68 (54.4%) women and 57 (45.6%) men. The average age of the participants was 32.1 years. The study group included 51 paramedics (40.8%), 39 (31.2%) nurses and 24 (19.2%) doctors. The remaining participants were 11 (8.8%). The socio-demographic data of the sample are presented in Table 1.

**Table 1:** Socio-demographic characteristics of the study sample (*N* = 125).

Parameter		Total (*N* = 125)
Sex	Female	68 (54.40%)
	Male	57 (45.60%)
Age (years)	Mean (SD)	32.11 (7.65)
	Median (quartiles)	30 (26–36)
	Range	23–60
Marital status	Single	34 (27.20%)
	In relation to	91 (72.80%)
Residence^a^	Country	27 (21.60%)
	City up to 50,000 inhabitants	18 (14.40%)
	City of 50,000–150,000 inhabitants	16 (12.80%)
	City of 150,000–500,000 inhabitants	23 (18.40%)
	City with more than 500,000 inhabitants	41 (32.80%)
Occupational group	Physiotherapist	5 (4.00%)
	Physician/dentist	24 (19.20%)
	Nurse	39 (31.20%)
	Midwife	5 (4.00%)
	Paramedic	51 (40.80%)
	Other	1 (0.80%)
Education	Secondary education	6 (4.80%)
	Bachelor’s degree	44 (35.20%)
	Master’s degree/medical doctor/dentist	68 (54.40%)
	PhD	7 (5.60%)
Seniority	Less than a year	6 (4.80%)
	1–5 years	72 (57.60%)
	6–10 years	19 (15.20%)
	11–15 years	12 (9.60%)
	16–20 years	7 (5.60%)
	More than 20 years	9 (7.20%)
Weekly working hours	20–39 h	18 (14.40%)
	40–59 h	57 (45.60%)
	60–79 h	38 (30.40%)
	80–99 h	9 (7.20%)
	100 h and more	3 (2.40%)
Place of employment	Hospital	86 (68.80%)
	Long-term care facilities	2 (1.60%)
	Primary health care	1 (0.80%)
	Others	36 (28.80%)
Works in shifts	No	24 (19.20%)
	Yes	101 (80.80%)
Type of hospital ward	Surgical	46 (36.80%)
	Non-surgical	25 (20.00%)
	Not applicable	54 (43.20%)
Working in more than one place	No	53 (42.40%)
	Yes	72 (57.60%)

a The adopted classification of place of residence is based on the population of each territorial unit, characteristic of Poland.

Based on the results, risk was identified for the three domains studied: hours of work, violence and substance abuse (Table 2). More than half of the subjects (52%) were identified as having an increased risk in the domain of working hours, which may mean that they work too much, in shift work, and this may directly affect the level of fatigue and work capacity. Close to half of those surveyed (49.6%) are at high risk of violence. More and more people working in the health care system are victims of patient abuse—this includes verbal and physical abuse. During the COVID-19 pandemic, this phenomenon has grown. Over half of respondents (52%) are at high risk of addiction. Based on the responses we can see that medical staff have easy access to psychoactive medicines - the prescriptions and the medicines themselves. Excessive workload, mental strain and physical fatigue can be predictors of the use of such stimulants, which of course can translate directly or indirectly into the safety of the patient and other medical personnel.

**Table 2:** Risk level results for each domain.

Risk domain	Risk level		
	No risk	Increased risk	High risk
Working hours	36 (28.80%)	65 (52.00%)	24 (19.20%)
Violence	18 (14.40%)	45 (36.00%)	62 (49.60%)
Abuse of psychoactive substances	20 (16.00%)	40 (32.00%)	65 (52.00%)

### 3.1. Bivariate analysis

A univariate analysis was performed to calculate the determinants of risk. The results of the analysis are as follows.

The risk in the domain of experiencing workplace violence is significantly higher in paramedics than in other professional groups. Additionally, nurses and midwives have experienced higher workplace violence than in representatives of “other” professions (Table 3). Paramedics are on the front line when it comes to contact with patients in hospital emergency departments or at the scene of an accident/patient’s home. Very often, patients waiting for their turn in a hospital emergency department become impatient and aggressive. Similar situations occur at the scene of an ambulance call. It also turns out that the odds of experiencing workplace violence is significantly higher in men than in women (Table 4).

**Table 3:** Influence of occupational groups on the risk scores in three domains: working hours, violence and abuse of psychoactive substances.

Risk domain	Occupational group	*N*	Mean	SD	Median	Min	Max	Q1	Q3	*p*
Working hours	Nurse/midwife	44	8.14	3.27	9.0	2	14	4.75	10.25	*p* = 0.531
	Physician/dentist	24	6.96	3.50	8.5	1	12	3.75	10.00	
	Paramedic	51	7.55	2.97	8.0	2	14	5.50	9.50	
	Other	6	7.50	2.51	7.0	5	12	6.00	8.00	
Violence	Nurse/midwife—A	44	9.25	3.71	10.0	0	14	7.75	12.25	*p* = 0.002^a^
	Physician/dentist—B	24	9.29	2.97	10.0	2	15	7.75	12.00	C > B, A, D A > D
	Paramedic—C	51	10.94	2.64	12.0	3	15	9.50	13.00	
	Other—D	6	4.00	5.18	3.0	0	14	0.75	3.75	
Abuse of psychoactive substances	Nurse/midwife	44	9.02	3.93	10.0	0	14	7.00	12.00	*p* = 0.55
	Physician/dentist	24	9.58	4.40	11.5	0	14	7.75	12.25	
	Paramedic	51	9.47	3.40	11.0	0	14	8.00	12.00	
	Other	6	8.50	2.95	9.0	4	11	7.00	11.00	

*p*, Kruskal–Wallis test + post-hoc analysis (Dunn’s test); SD, standard deviation; Q1, lower quartile; Q3, upper quartile.

a Statistically significant difference (*p* < 0.05).

**Table 4:** Influence of sex on the risk scores in three domains: working hours, violence and abuse of psychoactive substances.

Risk domain	Sex	*N*	Mean	SD	Median	Min	Max	Q1	Q3	*p*
Working hours	Female	68	7.85	3.28	9.0	2	14	4.00	10.00	*p* = 0.219
	Male	57	7.39	3.02	8.0	1	14	5.00	9.00	
Violence	Female	68	8.81	3.86	9.0	0	15	7.00	12.00	*p* = 0.003^a^
	Male	57	10.75	2.82	12.0	3	14	10.00	12.00	
Abuse of psychoactive substances	Female	68	9.29	3.94	11.0	0	14	7.75	12.00	*p* = 0.688
	Male	57	9.28	3.53	11.0	0	14	8.00	12.00	

*p*, Mann–Whitney test; SD, standard deviation; Q1, lower quartile; Q3, upper quartile.

a Statistically significant difference (*p* < 0.05).

We examined the relationship between highest educational attainment and experiencing workplace violence. The results show that the chance is significantly higher in those with a bachelor’s or master’s degree than in those with a high school education. This may be due to the fact that people with higher education tend to work in several places, such as paramedics and doctors. Medical workers with bachelor degree are the top risk of experiencing work place violence and abusing substances (Table 5), taking into account that most, paramedics make up of a great majority of staff with bachelor degrees.

**Table 5:** Influence of education on the risk scores in three domains: working hours, violence and abuse of psychoactive substances.

Risk domain	Education	*N*	Mean	SD	Median	Min	Max	Q1	Q3	*p*
Working hours	Secondary education—A	6	4.00	2.19	4.0	2	8	2.50	4.00	*p* = 0.015^a^
	Bachelor’s degree—B	44	8.34	3.06	8.0	2	14	6.00	11.00	B, C > A
	Master’s degree/medical doctor/dentist—C	68	7.62	3.15	8.0	1	14	5.00	10.00	
	PhD—D	7	6.57	2.51	8.0	2	8	6.00	8.00	
Violence	Secondary education—A	6	7.50	4.72	8.0	0	14	5.75	9.50	*p* = 0.026^a^
	Bachelor’s degree—B	44	10.57	3.11	12.0	1	15	9.00	13.00	B > D
	Master’s degree/medical doctor/dentist—C	68	9.66	3.44	10.0	0	15	8.00	12.00	
	PhD—D	7	6.43	4.20	6.0	2	12	3.00	9.50	
Abuse of psychoactive substances	Secondary education—A	6	4.00	3.52	2.0	1	9	2.00	6.50	*p* = 0.009^a^
	Bachelor’s degree—B	44	9.82	3.25	11.0	3	14	8.00	12.00	B, C > A
	Master’s degree/medical doctor/dentist—C	68	9.63	3.56	11.0	0	14	8.00	12.00	
	PhD—D	7	7.14	5.11	8.0	0	12	4.00	11.00	

*p*, Kruskal–Wallis test + post-hoc analysis (Dunn’s test); SD, standard deviation; Q1, lower quartile; Q3, upper quartile.

a Statistically significant difference (*p* < 0.05).

The study also examined the impact of weekly workload by using numbers of hours devoted to professional work. It found that the risk in the domain of violence is significantly higher in the group working 40–59 or 80 or more hours/week than in the group working 20–39 h/week. Additionally, the risk is significantly higher in the group working 60–79 h/week than in the group working less than 60 h/week (Table 6). Which can be deduced from the result that the high working hours is associated with the higher risk of experiencing violence, which can be associated with a higher likelihood of contact with a violent patient.

**Table 6:** Influence of weekly working hours on the risk scores in three domains: working hours, violence and abuse of psychoactive substances.

Risk domain	Weekly working hours	*N*	Mean	SD	Median	Min	Max	Q1	Q3	*p*
Working hours	20–39 h	18	5.67	3.56	3.5	2	12	3.00	9.00	*p* = 0.065
	40–59 h	57	8.12	2.61	8.0	1	13	7.00	10.00	
	60–79 h	38	7.68	3.04	8.0	2	14	5.00	10.00	
	80 h and more	12	8.17	4.37	7.5	2	14	4.00	12.25	
Violence	20–39 h—A	18	6.22	3.73	6.5	0	14	3.25	8.75	*p* < 0.001^a^
	40–59 h—B	57	9.12	3.38	10.0	0	15	8.00	11.00	D, B > A C > B, A
	60–79 h—C	38	11.89	2.01	12.0	6	15	11.25	13.00	
	80 h and more—D	12	10.67	3.11	12.0	5	14	8.00	13.25	
Abuse of psychoactive substances	20–39 h	18	8.28	4.86	10.5	0	13	4.50	12.50	*p* = 0.287
	40–59 h	57	9.98	3.30	11.0	0	14	8.00	12.00	
	60–79 h	38	9.26	3.27	10.0	0	13	8.00	11.00	
	80 h and more	12	7.58	4.81	8.5	0	13	2.00	11.25	

*p*, Kruskal–Wallis test + post-hoc analysis (Dunn’s test); SD, standard deviation; Q1, lower quartile; Q3, upper quartile.

a Statistically significant difference (*p* < 0.05).

Working extra hours, experiencing work violences, and substance abuse are significantly higher among shift workers and those HCWs who work in more than one place (Tables 7, 8).

**Table 7:** Influence of shift work on the risk scores in three domains: working hours, violence and abuse of psychoactive substances.

Risk domain	Shift work	*N*	Mean	SD	Median	Min	Max	Q1	Q3	*p*
Working hours	No	24	5.79	2.83	5.5	2	12	3.75	8	*p* = 0.001^a^
	Yes	101	8.08	3.08	8.0	1	14	6.00	10	
Violence	No	24	6.25	4.46	6.5	0	14	2.75	9	p < 0.001^a^
	Yes	101	10.51	2.75	11.0	3	15	9.00	13	
Abuse of psychoactive substances	No	24	7.08	4.90	7.0	0	13	3.25	12	*p* = 0.021^a^
	Yes	101	9.81	3.23	11.0	0	14	8.00	12	

*p*, Mann–Whitney test; SD, standard deviation; Q1, lower quartile; Q3, upper quartile.

a Statistically significant difference (*p* < 0.05).

**Table 8:** Influence of working on more than one place on the risk scores in three domains: working hours, violence and abuse of psychoactive substances.

Risk domain	Working in more than one place	*N*	Mean	SD	Median	Min	Max	Q1	Q3	*p*
Working hours	No	53	6.47	2.80	6	2	12	4.00	8	*p* < 0.001^a^
	Yes	72	8.50	3.15	9	1	14	7.00	11	
Violence	No	53	9.38	3.15	10	1	15	8.00	12	*p* = 0.108
	Yes	72	9.93	3.83	11	0	15	8.00	13	
Abuse of psychoactive substances	No	53	8.34	3.87	9	0	14	6.00	11	*p* = 0.006^a^
	Yes	72	9.99	3.52	11	0	14	8.00	12	

*p*, Mann–Whitney test; SD, standard deviation; Q1, lower quartile; Q3, upper quartile.

a Statistically significant difference (*p* < 0.05).

### 3.2. Multivariate analyses

Multivariate analyses were performed on variables that had a significant effect on a given risk domain in univariate analyses or were close to significance (i.e., had *p* < 0.1) and occupational group, which is the main variable of this analysis.

#### 3.2.1. Risk score in the domain: working hours

The multivariate linear regression model showed that the significant (*p* < 0.05) independent predictors of risk in working hours domain are (Table 9):

- Bachelor’s degree: the regression parameter is 5.023, so it raises the risk by an average of 5.023 points relative to secondary education;
- Master’s degree/doctor/dentist: the regression parameter is 4.545, so it raises the risk by an average of 4.545 points relative to secondary education;
- Weekly working hours of 80 h or more: the regression parameter is 2.263, so it raises the risk by 2.263 points on average relative to working less than 40 h/week;
- Working in shifts: the regression parameter is 2.099, so it raises the risk by 2.099 points on average;
- Working in more than one place: the regression parameter is 1.936, so it raises the risk by 1.936 points on average.

**Table 9:** Multivariate analysis—working hours domain.

Feature		Parameter	95% CI		*p*
Occupational group	Nurse/midwife	Ref.			
	Physician/dentist	−0.342	−1.932	1.248	0.674
	Paramedic	−0.05	−1.499	1.399	0.946
	Other	1.161	−1.785	4.106	0.442
Education	Secondary education	Ref.			
	Bachelor’s degree	5.023	2.434	7.613	<0.001^a^
	Master’s degree/medical doctor/dentist	4.545	1.939	7.152	0.001^a^
	PhD	3.296	0.016	6.576	0.051
Weekly working hours	20–39 h	Ref.			
	40–59 h	0.973	−0.625	2.571	0.235
	60–79 h	0.696	−0.936	2.328	0.405
	80 h and more	2.263	0.088	4.438	0.044^a^
Workplace	Hospital	Ref.			
	Other	−1.106	−2.555	0.342	0.137
Shift work	No	Ref.			
	Yes	2.099	0.42	3.778	0.016^a^
Working in more than one place	No	Ref.			
	Yes	1.936	0.913	2.958	<0.001^a^

*p*, multivariate linear regression.

a Relationship statistically significant (*p* < 0.05).

#### 3.2.2. Risk score in the domain: violence

An analogous analysis to that for working hours domain was conducted for violence domain (Table 10). The multivariate linear regression model showed that the significant (*p* < 0.05) independent predictors of risk in this domain are:

- Bachelor’s degree: the regression parameter is 3.538, so it raises the risk by an average of 3.538 points relative to secondary education;
- Master’s degree/doctor/dentist: the regression parameter is 3.235, so it raises the risk by an average of 3.235 points relative to secondary education;
- Job tenure of 6–10 years: the regression parameter is 3.257, so it raises the risk by an average of 3.257 points relative to tenure of less than 1 year;
- Seniority 11–15 years: the regression parameter is 3.542, so it raises the risk by an average of 3.542 points relative to seniority of less than a year;
- Weekly working hours 40–59 h: the regression parameter is 1.674, so it raises the risk by 1.674 points on average relative to working less than 40 h/week;
- Weekly working time of 60–79 h: the regression parameter is 4.001, so it raises the risk by an average of 4.001 points relative to working less than 40 h/week;
- Weekly working hours of 80 h or more: the regression parameter is 3.667, so it raises the risk by an average of 3.667 points relative to working less than 40 h/week;
- Shift work: the regression parameter is 3.098, so it raises the risk by an average of 3.098 points.

**Table 10:** Multivariate analysis—violence domain.

Feature		Parameter	95% CI		*p*
Occupational group	Nurse/midwife	Ref.			
	Physician/dentist	1.378	−0.163	2.92	0.083
	Paramedic	1.073	−0.183	2.329	0.097
	Other	−1.378	−4.155	1.398	0.333
Sex	Female	Ref.			
	Male	0.294	−0.824	1.413	0.607
Education	Secondary education	Ref.			
	Bachelor’s degree	3.538	1.021	6.054	0.007^a^
	Master’s degree/medical doctor/dentist	3.235	0.697	5.773	0.014^a^
	PhD	0.884	−2.2	3.969	0.575
Seniority	Less than a year	Ref.			
	1–5 years	1.935	−0.299	4.17	0.093
	6–10 years	3.257	0.83	5.684	0.01^a^
	11–15 years	3.542	0.837	6.246	0.012^a^
	16–20 years	1.402	−1.738	4.542	0.384
	More than 20 years	2.907	−0.397	6.212	0.087
Weekly working hours	20–39 h	Ref.			
	40–59 h	1.674	0.2	3.147	0.028^a^
	60–79 h	4.001	2.46	5.542	<0.001^a^
	80 h and more	3.667	1.572	5.762	0.001^a^
Shift work	No	Ref.			
	Yes	3.098	1.278	4.918	0.001^a^

*p*, multivariate linear regression.

a Relationship statistically significant (*p* < 0.05).

#### 3.2.3. Risk score in the domain: abuse of psychoactive substances

The last risk domain analyzed was psychoactive drug abuse. A multivariate linear regression model showed that significant (*p* < 0.05) independent predictors of risk in this domain are:

- Practicing a medical/dental profession: the regression parameter is 2.11, so it raises the risk by an average of 2.11 points relative to that of a nurse/midwife;
- Bachelor’s degree: the regression parameter is 5.358, so it raises the risk by an average of 5.358 points relative to secondary education;
- Master’s degree/doctor/dentist: the regression parameter is 5.217, so it raises the risk by an average of 5.217 points relative to secondary education;
- shift work: the regression parameter is 3.681, so it raises the risk by 3.681 points on average.
- Working in more than one place: the regression parameter is 1.87, so it raises the risk by 1.87 points on average.

## 4. Discussion

Healthcare system is fraught with many psychosocial risk factors. It is important to improve psychosocial ergonomics of the healthcare system to increase the preparedness for high demand situations like pandemics. Poor psychosocial risk factors has detrimental consequences on patient safety and workers mental health. In this study we measured outcomes of current healthcare system ergonomics on HCWs during COVID-19 pandemic in three dimensions of risk factors from the psychosocial group (see Table 11).

**Table 11:** Multivariate analysis—abuse of psychoactive substances domain.

Feature		Parameter	95% CI		*p*
Occupational group	Nurse/midwife	Ref.			
	Physician/dentist	2.11	0.236	3.984	0.029^a^
	Paramedic	0.677	−0.719	2.072	0.344
	Other	2.641	−0.73	6.012	0.127
Education	Secondary education	Ref.			
	Bachelor’s degree	5.358	2.56	8.156	<0.001^a^
	Master’s degree/medical doctor/dentist	5.217	2.397	8.037	<0.001^a^
	PhD	2.822	−0.965	6.61	0.147
Shift work	No	Ref.			
	Yes	3.681	1.754	5.607	<0.001^a^
Working in more than one place	No	Ref.			
	Yes	1.87	0.661	3.08	0.003^a^

*p*, multivariate linear regression.

a Relationship statistically significant (*p* < 0.05).

The labor system adopted in Poland (governed by the Labor Code) assumes that in the case of contract work, an employee works 7 h 25 min each day (for 5 days a week), or comes on duty for 12 h—which in the weekly calculation is supposed to give 40 h of work. In the case of employees who are employed on contractual agreements (running a sole proprietorship) it is different, because they can work more than 40 h a week—the labor code does not apply to them. The WHO is warning that long working hours contribute to deaths from stroke and ischemic heart disease. This mode of work is classified as high occupational risk (20). Medical personnel often work more than 40 h a week in Poland. In addition, they work shifts (80.8% of the respondents), and more than half of the respondents take on additional work (57.6%). This practice is common in many countries around the world, as confirmed by numerous scientific reports and reports (21, 22). More than half of those surveyed were found to have elevated hourly occupational risks. It should be noted that the results obtained in the study showed a higher occupational risk in the **domain** of working hours for those with higher than secondary education. A similar relationship was shown in a study conducted in medical workers in the Brazilian health care system (23). In Australia, on the other hand, medical students—young doctors—are working shorter hours than they were a few years ago. This has resulted in better patient safety and higher workers satisfaction (24). Working beyond the norm among medical personnel is nothing new. It is the result of many factors—including unsatisfactory salaries, staff shortages and attempts to “patch up” schedules. However, this is a special professional group, burdened with great responsibility for the health and lives of patients. Fatigue caused by overwork can have disastrous nipples, and this should not be forgotten.

Violence in the healthcare system is increasing. Upset patients, stresses of illness or loss of loved ones are among risk factors. Emergency department workers including doctors, nurses and paramedics are the most vulnerable group to work violences (25). This is a special field of medicine where the level of aggression from patients is high. Our study shows that HCWs with higher education and a high working hour are at higher risk of experiencing violence. Additionally, Shift workers are at the risk of experiencing violence by medical staff (26). Violence reported during night shift mor than day shifts, when workloads are higher, and less staff are available. A study of Chinese hospitals found that shift work is associated with the experience of violence (for example: verbal violence from patients, physical and psychological violence from patients and their families) by medical staff and doctors were more susceptible to experiencing non-physical violences (26). The problem of violence experienced by medical staff is very serious. In addition to the obvious issues like experiencing physical recognition from the patient, one must remember the psychological trauma, which can be profound and which can lead to a wave of departure from the profession. It is also necessary to look for answers as to why patients are violent - it is not always due to their condition. Often this aggression arises from a malfunctioning health care system, waiting too long for help and powerlessness. Policymakers need to keep this in mind as they put their medics on the front lines.

The psychophysical burden on medical personnel can make them to use substances as coping mechanism (27). A detailed multivariate analysis showed that doctors are more likely to use psychoactive drugs than nurses. In addition, higher education or shift work and working in more than one place are associated with higher substance abuse. That the problem is po-important is evidenced by statistics—about 20% of nursing staff have a problem with psychoactive drug abuse (28). A study in India found that more than 30% of resident doctors have a problem with psychoactive drug abuse (29). Substance abuse by medical personnel can also have many underpinnings and is the product of many factors. Working with patients and fighting for their health and life is very physically and mentally exhausting. Lack of alternatives to cope with such a heavy workload can result in a desire to turn to intoxicating substances. Once again, the role of prevention should be noted - it is also crucial in the middle of the work.

The results of our own research as well as demographic forecasts indicate that, with the current shortage of medical personnel, it is necessary to take multifaceted measures. One such task—for which employers are responsible—is the prevention of occupational risk reduction. Without the active participation of employers, managers and universities training future medics, it will not be possible to meet the growing expectations for nurses or doctors. This is a space to be exploited by public health specialists, who can develop and implement programs in the field of prevention of coping with stress, occupational burnout. Much of the responsibility lies with the employer, as he is the one who must see the need for such action—unless there are legal provisions in the public space forcing such action.

## 5. Conclusion

We carried out this study by investigating the HCWs of one of the most prestigious medical hospitals in Poland. Our findings show that the present psychosocial ergonomics of the Polish health system must be improved. The COVID-19 pandemic has been a compelling test to assess the current state. Our findings highlighted the fact that HCWs often worked overtime and that many cases of workplace violence and substance abuse were reported. This outcome may be used by health facilities, including hospitals and clinics, as a guideline to identify domains where intervention is required to address occupational risks. Medical personnel are an occupational group for whom preventive programmes should be devoted to improving ergonomics in the workplace. The lack of action in the above-mentioned domain is likely to increase the shortage of medical staff in the future, precisely because of inadequate work ergonomics.

### 5.1. Study limitation

The study has several limitations that must be taken into account when transposing the results and conclusions. First of all, it is important to emphasize the fact that the study group is not homogeneous, and consists of representatives of different medical professions, in varying numbers. When relating the results to individual professional groups, this should be taken into account, and inferences should be made with great caution. As the survey was conducted online and the survey link was distributed to professional groups, it is not possible to determine the return rate.

## Data availability statement

The raw data supporting the conclusions of this article will be made available by the authors, without undue reservation.

## Ethics statement

The study was carried out in accordance with the tenets of the Declaration of Helsinki and guidelines of Good Clinical Practices (World Medical Association, 2013). The study was fully anonymous and voluntary. Written informed consent for participation was not required for this study in accordance with the national legislation and the institutional requirements.

## Author contributions

ŁR and AK: conceptualization. ŁR: methodology, software, formal analysis, and supervision. ŁR and HS: validation. PG: investigation. ŁR and PG: resources. ŁR, IW, and HS: data curation and writing—original draft preparation. ŁR, AK, and HS: writing—review and editing. ŁR and AH-S: visualization and project administration. ŁR, IW, AK, and PG: funding acquisition. All authors contributed to the article and approved the submitted version.

## Funding

This work was funded from internal resources: SUBZ.E260.23.023 and CSM limit. The study was fully anonymous and voluntary.

## Conflict of interest

The authors declare that the research was conducted in the absence of any commercial or financial relationships that could be construed as a potential conflict of interest.

## Publisher’s note

All claims expressed in this article are solely those of the authors and do not necessarily represent those of their affiliated organizations, or those of the publisher, the editors and the reviewers. Any product that may be evaluated in this article, or claim that may be made by its manufacturer, is not guaranteed or endorsed by the publisher.
